# Psychosocial and Neurobiological Vulnerabilities of the Hospitalized Preterm Infant and Relevant Non-pharmacological Pain Mitigation Strategies

**DOI:** 10.3389/fped.2021.568755

**Published:** 2021-10-25

**Authors:** Ilana Shiff, Oana Bucsea, Rebecca Pillai Riddell

**Affiliations:** ^1^Department of Psychology, York University, Toronto, ON, Canada; ^2^Department of Psychiatry Research, Hospital for Sick Children, Toronto, ON, Canada; ^3^Department of Psychiatry, University of Toronto, Toronto, ON, Canada

**Keywords:** pain, preterm, neonate, non-pharmacological, pain management, neonatal intensive care unit (NICU)

## Abstract

**Background:** Preterm pain is common in the Neonatal Intensive Care Unit (NICU), with multiple invasive procedures occurring daily.

**Objective:** To review the psychosocial and neurobiological vulnerabilities of preterm infants and to provide an updated overview of non-pharmacological strategies for acute procedural pain in hospitalized preterm infants.

**Methods:** We utilized a narrative review methodology, which also included a synthesis of key pieces of published systematic reviews that are relevant to the current work.

**Results and Conclusions:** Preterm infants are uniquely susceptible to the impact of painful procedures and prolonged separation from caregivers that are often inherent in a NICU stay. Non-pharmacological interventions can be efficacious for mitigating procedural pain for preterm infants. Interventions should continue to be evaluated with high quality randomized controlled trials, and should endeavor to take into account the neurobiological and psychosocial aspects of preterm vulnerability for pain prevention and management strategies.

## Introduction

Pain in the preterm infant is a widespread challenge. The current estimated incidence rate of preterm birth is ~10.6% (1). Being born premature is the leading cause of mortality and one of the top causes of morbidity for children under the age of five (2, 3). Prematurity often results in extended hospitalizations in Neonatal Intensive Care Units (NICU) and adverse consequences for both children and their families, long after the children have left the hospital (3). Understanding the long-term implications of prematurity requires a comprehensive and integrated understanding of the young child's biopsychosocial development within the context of the NICU.

Biologically, premature infants struggle with multi-system challenges that result from a shortened developmental period *in utero*. Further, iatrogenic pain is a result of lifesaving procedures enacted to support the numerous challenges of prematurity (4). It is no longer debated that premature infants have the necessary peripheral and central anatomical architecture required for nociceptive transmission (5–7). In fact, their sensitive developing nervous systems have shown long-term changes in response to the pain and stress involved with preterm hospitalizations (e.g., reduced white matter microstructure and subcortical gray matter, dorsal horn central desensitization) (8).

In terms of social development, research has strongly supported the supposition that children are born seeking attachment to a primary caregiver and the development of this attachment relationship is a critical milestone on which future physical, cognitive, and emotional development is predicated (9). The requirements of the NICU severely redefine how this attachment relationship is built. Prolonged hospitalization often necessitates that the infant is physically distant from the primary caregiver and nuclear family. Further, the ability to receive physical contact is complicated by infant health conditions/interventions, competing familial demands of the caregiver, and physical/emotional strains on caregivers (10). However, promising evidence is being conducted to mitigate these social challenges through structured parental procedures (11, 12).

Unsurprisingly, given the biological and social implications of prematurity and its treatment, psychological implications also ensue. Children born premature have been shown to have altered cognitive and emotional development (e.g., language delays, cognitive delays, emotional, and behavioral development challenges) (13).

It is clear that clinicians and researchers must work from a transdisciplinary platform to best serve premature infants and their families. The current paper focuses on reviewing the biopsychosocial factors involved in preterm infant development as a means to contextualize professional understanding of non-pharmacological approaches to managing infant pain and mitigating the associated sequelae for both children and families.

It is critical to evaluate and develop pain management interventions, tailored to the unique physiological and psychosocial characteristics and needs of the preterm infant. This paper will first highlight the neurobiological, psychosocial, and contextual factors that contribute to the vulnerability of the preterm infant in the NICU. Using this framework, we will then present a narrative review of the recent studies on relevant non-pharmacological strategies for preterm infant procedural pain management.

### Pain Exposure in Hospitalized Preterm Infants

The last three decades of basic and applied neuroscience research has demonstrated that, contrary to prior medical dogma that cited the immature nervous system as evidence of the absence of nociceptive perception in infancy, nociceptive signals reach and are processed in the developing cortex of neonate (14). It is now known that neural pain transmission pathways are functioning as early as 22–24 weeks gestational age (15), with evidence of cortical responses to noxious stimulation as early as 25 weeks gestational age (16). Neonates not only possess the neuroanatomical and neurophysiological pathways required for nociceptive transmission, but it has been suggested that, as their descending inhibitory systems are functionally immature, they may experience increased sensitivity to pain compared to older children and adults (17, 18). This conclusion is based on the finding that neonates have lower thresholds for sensitization from noxious inputs resulting from the immaturity of their spinal cords, with earlier gestational ages linked to lower the pain thresholds (19). At early gestational ages, neonates may be experiencing non-noxious routine handling as painful until later in postnatal development (5). Finally, infants' emerging synaptic connectivity and network integration in their central nervous systems cause pain to be more diffuse and less spatially contained, potentially further increasing its impact.

The NICU experience for the preterm infant is rife with pain-inducing experiences. Routine painful procedures are common-place, with neonates experiencing on average 12–17 invasive procedures per day including heel sticks, endotracheal suctions, and intravenous line insertions (4, 20). Beyond this, many of the infants have underlying acute or chronic conditions, exposing them to a multitude of medical procedures and medications (3). Compounding the resulting exposure to pain, many preterm infants face these painful procedures without analgesic agents or behavioral pain management strategies, though practices vary across and within nations and interventions are improving due to increased attention (21).

#### Hospitalized Preterm Pain—Beyond a Simple Poke

The pain experienced by the hospitalized preterm infant is unique. While acute pain (a nociceptive pain with predictable duration caused by noxious stimulation) (22) and chronic pain (an often neuropathic pain lasting longer than 3 months) (23) have significant definitional consensus, the pain type exemplified in the NICU scenario is not as clearly defined (24). Though each invasive procedure alone constitutes one isolated painful experience, NICU infants can face repetitive painful procedures coupled with underlying medical conditions that can also cause pain. Preterm infants exhibit slower recovery from any one procedure as a result of their developing descending cortical control systems and developing nervous systems. This overlapping of acute pain experiences in the NICU has been referred to in the literature as a “chronically painful state,” as the pain of any one invasive procedure is exacerbated by the multitude of acute pain procedures experienced (8, 25). The effects of the pain exposure can accumulate, as the infants may not have had the chance to recover from one iatrogenic procedure before exposure to the next one (8, 25). Preterm infants' pain thresholds have been shown to be lowered from this repeated tactile stimulation and pain (i.e., sensitization) and they show an impaired ability to discriminate between noxious and non-noxious stimuli until 35 weeks gestation (5), potentially causing experiences such as diaper changes and bathing to be perceived as noxious (19).

Other research has shown that the more painful experiences neonates have, the more reactive they become to subsequent pain stimuli. Research has illustrated that reactivity to stimuli is directly associated with prior pain exposures, both in the previous 24 h and cumulatively since birth (26–28). Altogether, these experiences result in continuous activation of the internal physiological systems that are responsible for maintaining stability in response to stress (i.e., allostasis) (3, 29). While engagement of these systems is adaptive in the short-term, allostatic load occurs when the systems are overloaded due to continuous elevation over time (30), such as in response to continuous unmanaged painful procedures in the neonatal period (3). Allostatic load further exacerbates the chronicity of the pain and compromises infants' ability to adaptively respond to future stress, which can lead to negative long-term developmental and health outcomes (3). The complexity of an infant receiving multiple painful procedures of varying intensity and quantity has led a team of researchers to create a research measure to help quantify the pain load of individual infants (31). This measure was created to improve both the clinical and research understanding of pain in the hospitalized preterm by facilitating a more accurate description of the amount of pain burden a child has experienced over a prolonged period of time.

### Longer-Term Neuroanatomical, Neurophysiological, and Neurodevelopmental Outcomes Associated With Preterm Pain Exposure

As discussed, the immaturity of preterm infants' neurobiological systems likely increases their vulnerability to undermanaged pain experienced in the NICU, making them both more sensitive to pain and more susceptible to its detrimental effects (8, 18, 32). During the third trimester the microsystem of the fetal brain undergoes significant developmental changes, including the establishment, organization, and proliferation of cortical neurons; development of dendrites and axons; formation and pruning of synapses, axonal development in the cerebellum; and differentiation of glial cells (3, 33, 34). Animal studies have illustrated that brain development in the weeks immediately prior to and following birth, such as structural growth and synaptic formation, is especially significant (34). This has important implications for preterm infants as a high percentage of the invasive procedures they endure occur within the first week after birth (4, 20).

As a result of the rapid neurobiological changes that occur in the first weeks of life and the hypersensitivity of preterm infants' brain systems to stimulation (34), NICU experiences and the repetitive early pain exposure may compromise central nervous system development and result in structural and functional reorganization (32). Firstly, structural brain changes, such as reduced gray and white matter, have been observed throughout childhood and adolescence in those who were born very preterm (less than or equal to 32 weeks gestational age) (35–39). Delayed cortical maturation specifically, resulting in cortical thinness, is evident (40). Importantly, Vinall et al. sought to isolate the specific relationship between neonatal pain-related stress and altered cortical thickness, by adjusting for confounding factors such as gestational age, exposure to mechanical ventilation, morphine exposure, surgeries, and severity of illness and infection (41). Compared to their term-born peers, very preterm infants who experienced neonatal pain-related stress exhibit significantly thinner cerebral cortexes, particularly in the frontal and parietal lobes (41).

Further, neurophysiological systems and processes are also impacted (18). Firstly, repeated pain exposures, such as those experienced by hospitalized preterm infants, result in changes to nociceptive neural pathways (42, 43) as well as behavioral and neural hypersensitivity to later pain, known as hyperalgesia (44, 45). Hyperalgesia can occur both in the area of local tissue damage as well as elsewhere on the body; hospitalized preterm infants with tissue damage on one leg showed lower pain threshold on the unimpacted, contralateral foot (46). Secondly, short- and long-term changes to the neuroendocrine stress systems, in particular hypothalamic-pituitary-adrenal (HPA) axis activity, are exhibited in response to the stress of cumulative neonatal procedural pain (47, 48).

In addition to the structural and functional brain changes associated with cumulative pain exposure, early pain experiences impact neurodevelopmental outcomes, such as learning, cognition, and behavioral problems (19, 49). Research has demonstrated a positive correlation between the number of skin-breaking procedures that a preterm infant is exposed to and cognitive and motor outcomes later in infancy (50). Additionally, exposure to pain early in life can impact a child's cognitive outcomes at school-age, such as capacity for sustained attention and learning (51).

### Neuroprotective Developmental Care—The Importance of Parental Presence and Sensitivity for Preterm Infant Pain Responding and Outcomes

Understanding the psychosocial underpinnings of neonatal pain-related distress responding allows both clinicians and researchers to take a more nuanced approach to infant pain management. Human infants, born with an inherent need for social connection, are biologically predisposed to seek help from others in times of distress (52). Signaling caregivers is purported to be an evolutionary survival instinct (53). Research in older infants has shown that the intensity of infant pain-related signaling is significantly related to parent facial expressions during the pain experience and how the caregiver self-reports their approach to relationships (54, 55). While neonates lack the capacity to communicate with language, crying is a powerful communication tool that they can utilize (56). Infants are entirely dependent on others to both identify and tend to their needs, such as addressing their distress while in pain and serving as an external regulation system. Throughout the first years of life, as infants signal distress and caregivers respond (ideally to reduce the distress), the infant learns to build autonomous self-regulatory strategies. The attachment relationship, as measured by classic behavioral separation and reunion procedures, is predicated on how the infant learns to manage distress with their caregiver. The strength and quality of the attachment relationship, which begins forming at birth, is considered a strong predictor of many socio-emotional, academic, and behavioral outcomes (57).

Parents' contingent and sensitive responding to infant distress is considered a critical driver of the attachment relationship. The NICU context may complicate the development of attachment relationships, as defined by Bowlby (9). Hospitalized preterm infants may experience physical separation from their primary caregivers for many hours a day, while other non-parental caregivers provide feeding, handling, and other care duties. This physical separation limits the tactile and verbal social stimulation that infants require (58) and the comforting associations with just one primary caregiver. Moreover, Attachment Theory is predicated on contingent responding to distress of all kinds such as distress caused by pain, fear, hunger, and loneliness (9). When parents cannot be at the bedside around the clock, much of the soothing done in response to distressing events will be conducted by non-parental caregivers. However, these effects are not irreversible. Environmental enrichment, such as increased proximity of NICU infants to their caregivers and optimizing parental sensitivity in response to distress, is shown to reverse the effects in rodents (59). In human neonates, positive parent interactions have also been found to buffer the neurobiological (60), cognitive (61), and emotional (62) effects of neonatal stress and pain.

Family centered care (FCC) is a philosophical and practical approach which presents a set of guiding principles for pediatric healthcare settings. At the core of FCC is the promotion of partnership and collaboration with patients and their families in the delivery of healthcare and prioritization of the needs of the family in an effort to improve infant and family outcomes (63). FCC frames families as an integral part of the care team, empowering them as part of the decision-making process (64) and shifting focus away from disease and toward patients in the context of their families and communities (65). In the NICU, this practice is especially significant given the infants' needs within the context of the family and the proven psychological and developmental implications of family separation. NICU FCC can include family education, parental participation in clinical decision-making, involvement in caregiving, and facilitation of family visitation (65). A wide spectrum of family centered care models exist, and Frank and O'Brien recently developed a taxonomy for classifying the various interventions (63). FCC has been shown to have benefits such as decreased length of stay (66), improved parent satisfaction (67) and enhanced psychological well-being (68).

In addition to incorporating FCC principles, NICU staff should advise parents to physically soothe their preterm infants during painful procedures whenever possible. Contrary to commonly held misconceptions, infants will not associate the parents with distressing stimuli and their soothing will instead strengthen the infant-caregiver relationship. Researchers have speculated that early fear-conditioning, *via* the amygdala, ensures that infants do not make an avoidance connection to parents who respond to infant distress as doing so would jeopardize the infant (69). Recent studies have demonstrated that maternal skin-to-skin care may be neuroprotective as the brain is not as activated during painful procedures (70, 71). One study (71) compared three groups of parental holding [skin to skin holding, parent holding while clothed, no parent holding (child in developmentally-sensitive nesting/tucking while in cot)] and found that the parent holding while clothed group showed the most infant pain-related cortical activity. Authors speculated that due to sensitivity of the preterm to disruption, only skin-to-skin contact (not clothed parent holding) was powerful enough to overcome the disruption of moving the infant from the cot for a painful procedure.

### Non-pharmacological Management of Preterm Infant Procedural Pain

This paper has set out to contextualize the unique neurobiological stage of the hospitalized preterm infant within the caregiver context. Due to the unique neurophysiological and psychosocial needs of the preterm neonate outlined here, neonatal pain management cannot merely be a downward extension of the methods shown to be efficacious in older populations (17). Moreover, many strategies that have been shown to be efficacious in older infants, such as distraction (72), are simply not feasible in this population due to limited motor and cognitive capacity (52). What will now follow is a narrative review on non-pharmacological pain management strategies for acute procedural pain in preterm infants. The strategies presented reflect a nuanced understanding of the unique neurophysiological and psychosocial context of the pained preterm infant. Many require the participation of a caregiver to initiate/maintain a strategy, and are divided into minimal, moderate and significant caregiver involvement. When possible, reference will be made to the published results from a 2015 Cochrane Review entitled “Non-pharmacological management of infant and young child procedural pain” (72), relevant reviews [e.g., (73–75)], and new trials that we have found in a recent literature search.

## Review of Non-Pharmacological Procedural Pain Management Strategies

The review of evidence for non-pharmacological pain management strategies will be summarized in four different groupings. First as aforementioned, we will present strategies that parents and health professionals can enact that require differing levels of caregiver involvement during the enactment of the pain management strategy. We will then present evidence on the role of non-pharmacological pain management strategies as an additive on top of sucrose (one of the most commonly researched pharmacological strategies) or on top of another non-pharmacological strategy.

### Strategies Modifying the Context of the Painful Procedure

#### Light Reduction Interventions

These strategies involve minimizing the amount of light the infant is exposed to in order to lower their pain reactivity and stress, either by direct (i.e., covering their eyes) or indirect (i.e., placing a blanket over the infants' incubator) manipulation. Alemdar and Ozdemic found covering infants' eyes during venipuncture to be efficacious at reducing pain responding (76). Another randomized controlled trial by Alemdar demonstrated that covering infants' incubators during peripheral cannulation was also efficacious (77). Additional research on the impact of light reduction on preterm pain responding is required before conclusive statements about its efficacy are made, but the strategy shows promise.

#### Sound Addition Interventions

These strategies involve exposing infants to soothing sounds such as a reproduction of his or her mother's voice within the womb, designed to help simulate the soothing fetal environment. Results from the 2015 Cochrane Review did not find simulated mother's voice to be efficacious at reducing pain responding (72). More recently, a number of researchers have examined the impact of soothing sounds or music on preterm infant pain responding during various painful procedures.

Chen and Zhou examined the impact of maternal voice stimulation during heel blood collection, illustrating that it is efficacious at reducing behavioral and physiological pain responding (78). Chirico et al. also demonstrated a significant effect of recorded maternal voice for reducing behavioral pain responses during heel lance (79). On the other hand, Alemdar and Ozdemir did not find intrauterine sounds to significantly reduce pain scores during or after venipuncture (76). Alemdar also examined the impact of recorded maternal voice during peripheral cannulation, and found it was not significantly efficacious (77).

Music exposure is another strategy that researchers have examined the impact of for preterm pain responding. Exposure to classical music was found to be efficacious for reducing pain responding during heel lance (80), as was exposure to the music listened to by mothers during pregnancy (81). While studies have yielded mixed results, soothing sounds and music appear promising for reducing preterm infant pain responding.

#### Smell Addition Interventions

These strategies involve modifying the infants' environment by exposing them to a soothing smell during the procedure, such as their mother's odor, breastmilk odor, lavender, or vanilla odors. One randomized controlled trial examined the effects of breastmilk odor compared with vanilla odor and no odor, illustrating that breastmilk odor lowers pain responding both during and after venipuncture (82). Alemdar and Ozdemir found that various maternal smells, such as amniotic fluid, mother's milk, and mother's odor did not reduce infants' behavioral pain responding during heel stick (83). Breast milk odor also did not significantly reduce pain scores (77). Additional research on the impact of added smells with preterm neonates is warranted.

#### Multi-Sensory Bundle Interventions

These strategies modify the context in which the painful procedure takes place, combining both direct and indirect pain management strategies (i.e., soothing smells, low lights and noise, non-nutritive sucking, swaddling, etc.). The 2015 Cochrane Review found this type of strategy, labeled environmental modification, not to be efficacious for preterm infants' pain reactivity (within 30 s of the pain stimulus) but was efficacious for improving their immediate pain regulation (after the first 30 s) (72). An increasing number of studies are examining the combined effect of multiple non-pharmacological interventions. One group examined the combination of non-nutritive sucking and swaddling, demonstrating that it is more effective than standard care in alleviating preterm infant pain (84). Other researchers examined a bundle of various strategies, targeting multi-sensory stimulation, such as Behman Vashani et al. who compared multi-sensory stimulation (stimulation of taste, touch, sight, and smell) to standard care and found it to be significantly efficacious (85). Labeled as a developmental care bundle, another group found the combination of environmental modifications, positioning and containment, and oxygen supplementation to be more efficacious than standard care (86). Finally, a recent trial demonstrated the efficacy of the integration of breastmilk odor and taste, heart beat sounds, and NNS for preterm infants during venipuncture (87). While the combination of strategies employed differ, the emerging evidence suggests that multi-sensory bundle interventions are efficacious for preterm pain management.

### Strategies Requiring Caregivers to Initiate and/or Maintain Some Contact

#### Non-nutritive Sucking

This strategy involves the placing of an object (e.g., pacifier, non-lactating nipple) into an infant's mouth to stimulate oro-tactile or sucking behaviors. The 2015 Cochrane Review concluded that NNS was not found to be efficacious for relieving preterm infants' pain reactivity, but it was efficacious for improving immediate pain regulation (72). New evidence, however, suggests that compared with routine care, NNS significantly reduces behavioral pain scores and crying time both during heel stick and recovery (88). NNS is a well-established and validated approach for infant pain management, and further research can help elucidate whether it is impactful for initial pain reactivity in preterm infants.

#### Swallowing Water

This strategy involves administering water for ingestion without an instrument that would incite extensive sucking (e.g., a dropper). The 2015 Cochrane review demonstrated that swallowing water was not an efficacious strategy for preterm pain management (72) and it was further recommended to no longer explore this approach.

#### Swaddling

This strategy involves securely wrapping an infant in a blanket to contain the child's limbs comfortably to center. It was found to show promise for preterm infant pain management (72). A randomized controlled trial found that swaddled preterm infants exhibited lower pain scores and faster returns to baseline following blood sampling compared to un-swaddled infants (89). Another recent study also demonstrated that swaddling is efficacious at reducing behavioral and physiological indicators of pain (90). The evidence appears to support the use of swaddling to manage preterm infant pain.

### Strategies Incorporating Caregiver Contact

#### Facilitated Tucking

This strategy involves firmly containing the infant using a caregiver's hands on both head and lower limbs to maintain a “folded-in” or “tucked” position. Pillai Riddell et al. review deemed facilitated tucking to be an efficacious intervention for preterm pain management (72). Another review conducted by Hartley et al. also demonstrated the efficacy of facilitated tucking in this population (75). A recent review by Neto et al. showed that facilitated tucking is associated with a significant reduction in pain compared to routine care, but not compared to opioid or oral glucose administration (74). Following these reviews, a number of randomized controlled trials have examined the efficacy of facilitated tucking for preterm pain management. Ranjbar et al. found facilitated tucking to be less efficacious than oral dextrose in a crossover design during routine blood sampling, but more effective than routine care (91). Taplak and Bayat demonstrated that facilitated tucking was more efficacious than routine care at reducing behavioral pain scores before and after endotracheal suctioning, but not during the procedure (92). Cirik and Effy found that facilitated tucking did not significantly reduce pain during orogastric tube insertion in preterm infants (93). Overall, while there are mixed findings that are potentially due to the inclusion of different painful procedures and timepoints of pain measurement, facilitated tucking continues to be studied and often found to be efficacious.

#### Touch-Related Interventions

These strategies involve a variety of tactile-based interventions (e.g., traditional massage, Yakson therapeutic touch, stimulating a specific pressure point on the infant's body, and applying pressure to the site of the painful procedure) designed to provide physical counter-stimulation to the nociceptive input. Pillai Riddell et al. found that touch/massage-related interventions were efficacious at reducing pain reactivity but not for immediate pain regulation. A randomized controlled trial examining acupressure found that the technique did not reduce behavioral pain scores but did significantly reduce crying duration (94). However, a cross-over trial by Fatollahzade et al. demonstrated that gentle human touch during endotracheal suctioning significantly reduces pain scores (95). The mixed findings on this technique may be due to the variation of touch-related strategies that are captured in this category, and additional trials will enable future reviews to look at this category more parsimoniously.

#### Kangaroo or Skin-to-Skin Care

This strategy, otherwise known as skin-to-skin, involves the infant being placed upright on the mother's bare chest providing maximal contact between mother and infant. Twenty-five studies were included in the 2017 Cochrane Review by Johnston et al. (73). The randomized trials in this review were considered mainly to have low risk of bias, but this integrated special consideration that blinding of outcome assessors was not possible in that context. Both physiological and behavioral outcomes were included. Studies indicated that kangaroo care is efficacious in reducing pain responding initially and during recovery from painful procedures. Additional trials have since examined kangaroo care during various procedures, with one illustrating that kangaroo care did not reduce pain compared to standard care during preterm infants' eye examinations (96). Optimal duration of kangaroo care before, during, or after the procedures requires more research.

#### Breastfeeding/Breastmilk

This strategy entails either direct breastfeeding or administering supplemental breastmilk during painful medical procedures. A Cochrane Review on the topic illustrated the efficacy of breastfeeding at reducing physiological and behavioral pain responding. Supplemental breast milk, on the other hand, showed mixed and unconvincing results (88). Notably, the preterm infants included in that review were stable and relatively healthy. The preterm infants in the NICU may be incapable of direct breastfeeding and may benefit from placement of breastmilk on the tongue. Additional research examining breastfeeding/supplemental breastmilk in the hospitalized preterm infant is warranted.

### Additive Effect of Non-pharmacological Interventions

As mentioned earlier, the final group of non-pharmacological trials to be reviewed were trials that examined the additive effect of a non-pharmacological intervention. These types of randomized controlled trials could isolate the “additive” effect by their study design. This research is newly emerging and a result of the ethical shift regarding the acceptability of having a “no-treatment” control in pain management trials. Thus, the question shifts from “is the intervention better than no intervention?” to “does an intervention add significant benefit to another intervention?”.

#### Additive Effect of Non-pharmacological Interventions on a Non-pharmacological Intervention

This group of studies examined the additive effect of a variety of non-pharmacological interventions (i.e., maternal holding, exposure to warm temperatures, NNS, facilitated tucking, etc.) on another non-pharmacological intervention. One group of researchers illustrated that the addition of the combination of a lullaby and NNS on top of facilitated tucking and holding significantly reduced pain scores during heel lance (97). Another group showed that compared to NNS alone, the combination of NNS and mother's milk odor was associated with significantly lower behavioral pain scores during venipuncture and reduced crying after venipuncture (98). Finally, a trial comparing the efficacy of the combined use of facilitated tucking and NNS did not reduce pain during heel-stick significantly more than NNS alone, but was associated with faster recovery following the procedure (99). In addition to the ethical implications of administering a pain management intervention to all participants, combining non-pharmacological pain management appear to be more efficacious at reducing procedural pain responding in preterm infants than individual strategies.

#### Additive Effect of Non-pharmacological Interventions on Oral Sucrose

This group of studies examined the additive effect of a variety of non-pharmacological interventions (i.e., maternal holding, exposure to warm temperatures, NNS, facilitated tucking, etc.) on a sweet solution, such as sucrose. Campbell-Yeo et al. demonstrated the additive effect of twin co-bedding on sucrose for physiologic recovery from heel lance (100). The same research team examined the additive effect of kangaroo care on sucrose, demonstrating that they are equally effective at reducing stable preterm infants' distress responding to heel lance but that the combination did not provide additional benefit (101). Magnetic acupuncture on top of sucrose was examined during heel prick and found to be significantly more efficacious than sucrose alone (102). Focusing on repeated procedural pain exposure rather than single procedures, Gao et al. illustrated that the combination of NNS and sucrose was more efficacious than sucrose alone (88). Finally, moving away from skin-breaking procedures, the combination of pacifier and sucrose was shown to be optimally effective during feeding tube insertion (103). Given that sucrose is a common pain management agent for preterm procedural pain, it is important to note the additive benefit of non-pharmacological strategies that most of these trials have demonstrated.

## Discussion

Millions of children are born preterm every year. Medical advances in many middle and high income countries have exponentially increased survival, even *in situations* where the infant is born at just under 23 weeks (104). But their survival comes at the cost of exposure to invasive procedures to save their lives. Preterm infants are uniquely susceptible to the impact of invasive procedures and separation inherent in a NICU stay. It is critical to take into account the neurobiological and psychosocial development of a preterm child when considering options for pain management that use cognitive, behavioral, or context-dependent strategies. The state of the literature on non-pharmacological preterm pain management is continuously growing, with particularly increasing numbers of trials focusing on facilitated tucking and multi-sensory bundles. Clinicians and researchers alike can benefit from examining the breadth of studies and trends within the updated literature.

In terms of research, trialists should continue to conduct well-powered randomized controlled trials on the individual, combined, and additive effect of non-pharmacological interventions for preterm pain management. It is critical for trialists to clearly operationalize pain measurement epochs, the positioning of the child (e.g., who is holding child, details of how child is held or placed in cot), pharmacological factors at time of procedure, and provide clear data regarding the exact nature of the intervention being tested. While most journals have now made CONSORT standards of reporting mandatory, there are still many studies being published without adherence to these recommendations (http://www.consort-statement.org/). A developing positive trend in the literature is the trial structure which enables the examination of additive effects of pain management strategies, driven by the ethical challenges of using no-treatment control groups.

Clinicians are encouraged to incorporate caregivers in as many aspects of pain management as possible. The available evidence suggests that for preterm infants, non-nutritive sucking, facilitated tucking, swaddling, light reduction, multisensory bundle strategies and kangaroo care appear to be efficacious. Combining a non-pharmacological intervention with sucrose as well as two non-pharmacological interventions also appear to be promising ways to manage the iatrogenic pain inherent in a NICU stay. In general, proximity to a caregiver before, during, and after a procedure is ideal practice, and caregivers can be included in pain management strategy implementation when possible. Infants will not learn to associate the caregiver with distress, they will come to associate the caregiver with safety when distressed.

## Author Contributions

IS and RPR conceptualized the manuscript. IS wrote the first draft with guidance from RPR. IS and OB carried out the literature searching. IS, OB, and RPR edited and revised it. All authors approved the final manuscript for submission.

## Funding

This research is supported by a salary and operating grant award to RPR from the Canadian Institute of Health Research (CPG: 163988). Scholarship funding was awarded to IS from the Ontario Graduate Scholarship, HSBC Bank of Canada, and the Lillian Meighen Wright Maternal-Child Health Graduate Scholarship. Scholarship funding was awarded to OB from the Canadian Institute of Health Research (FBD–170829). IS and OB are trainee members of Pain in Child Health (PICH), a strategic research training initiative of the Canadian Institutes of Health Research.

## Conflict of Interest

The authors declare that the research was conducted in the absence of any commercial or financial relationships that could be construed as a potential conflict of interest.

## Publisher's Note

All claims expressed in this article are solely those of the authors and do not necessarily represent those of their affiliated organizations, or those of the publisher, the editors and the reviewers. Any product that may be evaluated in this article, or claim that may be made by its manufacturer, is not guaranteed or endorsed by the publisher.

## References

[1] Chawanpaiboon S Vogel JP Moller A-B Lumbiganon P Petzold M Hogan D. Global, regional, and national estimates of levels of preterm birth in 2014: a systematic review and modelling analysis. Lancet Glob Health. (2019) 7:e37–46. 10.1016/S2214-109X(18)30451-0 30389451 PMC6293055

[2] Lee AC Blencowe H Lawn JE. Small babies, big numbers: global estimates of preterm birth. Lancet Glob Health. (2019) 7:e2–3. 10.1016/S2214-109X(18)30484-4 30389450

[3] Grunau RE Holsti L Peters JW. Long-term consequences of pain in human neonates. In: Tibboel D Bhat R editors. Seminars in Fetal and Neonatal Medicine. Amsterdam: Elsevier (2006). p. 268–75. 10.1016/j.siny.2006.02.007 16632415

[4] Carbajal R Rousset A Danan C Coquery S Nolent P Ducrocq S. Epidemiology and treatment of painful procedures in neonates in intensive care units. Jama. (2008) 300:60–70. 10.1001/jama.300.1.60 18594041

[5] Fabrizi L Slater R Worley A Meek J Boyd S Olhede S. A shift in sensory processing that enables the developing human brain to discriminate touch from pain. Curr Biol. (2011) 21:1552–8. 10.1016/j.cub.2011.08.010 21906948 PMC3191265

[6] King TE Barr GA. Spinal cord ionotropic glutamate receptors function in formalin-induced nociception in preweaning rats. Psychopharmacology. (2007) 192:489–98. 10.1007/s00213-007-0735-x 17356878

[7] Ruda MA Ling Q-D Hohmann AG Peng YB Tachibana T. Altered nociceptive neuronal circuits after neonatal peripheral inflammation. Science. (2000) 289:628–30. 10.1126/science.289.5479.628 10915627

[8] DiLorenzo M Pillai Riddell R Holsti L. Beyond acute pain: understanding chronic pain in infancy. Children. (2016) 3:26. 10.3390/children3040026 27834860 PMC5184801

[9] Bowlby J. Attachment and Loss: Vol. 1. 1 Attach. New York, NY: Basic Books (1969).

[10] Pineda R Bender J Hall B Shabosky L Annecca A Smith J. Parent participation in the neonatal intensive care unit: predictors and relationships to neurobehavior and developmental outcomes. Early Hum Dev. (2018) 117:32–8. 10.1016/j.earlhumdev.2017.12.008 29275070 PMC5856604

[11] Filippa M Poisbeau P Mairesse J Monaci MG Baud O Huppi P. Pain, parental involvement, and oxytocin in the neonatal intensive care unit. Front Psychol. (2019) 10:715. 10.3389/fpsyg.2019.00715 31001173 PMC6454868

[12] Arnon S Diamant C Bauer S Regev R Sirota G Litmanovitz I. Maternal singing during kangaroo care led to autonomic stability in preterm infants and reduced maternal anxiety. Acta Paediatr. (2014) 103:1039–44. 10.1111/apa.12744 25039678

[13] Woodward LJ Moor S Hood KM Champion PR Foster-Cohen S Inder TE. Very preterm children show impairments across multiple neurodevelopmental domains by age 4 years. Arch Dis Child Fetal Neonatal Ed. (2009) 94:339–44. 10.1136/adc.2008.146282 19307223

[14] Fitzgerald M Millard C Macintosh N. Hyperalgesia in premature infants. Lancet. (1988) 331:292. 10.1016/S0140-6736(88)90365-0 2893095

[15] Byers JF Thornley K. Cueing into infant pain. MCN Am J Matern Nurs. (2004) 29:84–9. 10.1097/00005721-200403000-00004 15028913

[16] Slater R Cantarella A Gallella S Worley A Boyd S Meek J. Cortical pain responses in human infants. J Neurosci. (2006) 26:3662–6. 10.1523/JNEUROSCI.0348-06.2006 16597720 PMC6674141

[17] Fitzgerald M Walker SM. Infant pain management: a developmental neurobiological approach. Nat Clin Pract Neurol. (2009) 5:35–50. 10.1038/ncpneuro0984 19129789

[18] Fitzgerald M. The development of nociceptive circuits. Nat Rev Neurosci. (2005) 6:507–20. 10.1038/nrn1701 15995722

[19] Grunau RE. Early pain in preterm infants: a model of long-term effects. Clin Perinatol. (2002) 29:373–94. 10.1016/S0095-5108(02)00012-X 12380464

[20] Cruz MD Fernandes AM Oliveira CR. Epidemiology of painful procedures performed in neonates: a systematic review of observational studies. Eur J Pain. (2016) 20:489–98. 10.1002/ejp.757 26223408

[21] Committee on Fetus and Newborn and Section on Anesthesiology and Pain Medicine. Prevention and management of procedural pain in the neonate: an update. Pediatrics. (2016) 137:e20154271. 10.1542/peds.2015-4271 26810788

[22] Schug SA. 2011–The Global Year Against Acute Pain. London: SAGE Publications Sage UK (2011).

[23] Treede R-D Rief W Barke A Aziz Q Bennett MI Benoliel R. Chronic pain as a symptom or a disease: the IASP Classification of Chronic Pain for the: international classification of diseases (ICD-11). Pain. (2019) 160:19–27. 10.1097/j.pain.0000000000001384 30586067

[24] Anand KJS. Defining pain in newborns: need for a uniform taxonomy? Acta Paediatr. (2017) 106:1438–44. 10.1111/apa.13936 28556311 PMC5601230

[25] Pillai Riddell R Stevens BJ McKeever P Gibbins S Asztalos L Katz J. Chronic pain in hospitalized infants: health professionals' perspectives. J Pain. (2009) 10:1217–25. 10.1016/j.jpain.2009.04.013 19541547

[26] Grunau RE Holsti L Whitfield MF Ling E. Are twitches, startles, and body movements pain indicators in extremely low birth weight infants? Clin J Pain. (2000) 16:37–45. 10.1097/00002508-200003000-00007 10741817

[27] Porter FL Wolf CM Miller JP. The effect of handling and immobilization on the response to acute pain in newborn infants. Pediatrics. (1998) 102:1383–9. 10.1542/peds.102.6.1383 9832573

[28] Grunau RE Oberlander TF Whitfield MF Fitzgerald C Lee SK. Demographic and therapeutic determinants of pain reactivity in very low birth weight neonates at 32 weeks' postconceptional age. Pediatrics. (2001) 107:105–12. 10.1542/peds.107.1.105 11134442

[29] Casavant SG Cong X Fitch RH Moore J Rosenkrantz T Starkweather A. Allostatic load and biomarkers of stress in the preterm infant: an integrative review. Biol Res Nurs. (2019) 21:210–23. 10.1177/1099800418824415 30654634

[30] McEwen BS. Protection and damage from acute and chronic stress: allostasis and allostatic overload and relevance to the pathophysiology of psychiatric disorders. Ann N Y Acad Sci. (2004) 1032:1–7. 10.1196/annals.1314.001 15677391

[31] Laudiano-Dray MP Pillai Riddell R Jones L Iyer R Whitehead K Fitzgerald M. Quantification of neonatal procedural pain severity: a platform for estimating total pain burden in individual infants. Pain. (2020) 161:1270–7. 10.1097/j.pain.0000000000001814 31977932

[32] Grunau RE. Neonatal pain in very preterm infants: long-term effects on brain, neurodevelopment and pain reactivity. Rambam Maimonides Med J. (2013) 4:4 10.5041/RMMJ.10132 24228168 PMC3820298

[33] Volpe JJ. Brain injury in premature infants: a complex amalgam of destructive and developmental disturbances. Lancet Neurol. (2009) 8:110–24. 10.1016/S1474-4422(08)70294-1 19081519 PMC2707149

[34] Ranger M Grunau RE. Early repetitive pain in preterm infants in relation to the developing brain. Pain Manag. (2014) 4:57–67. 10.2217/pmt.13.61 24641344 PMC3975052

[35] Anand KJ. From the gate-control theory to brain programs for neonatal pain. In: Buonocore G Bellieni CV editors. Neonatal Pain. Milano: Springer (2008). p. 141–7.

[36] Nosarti C Mechelli A Herrera A Walshe M Shergill SS Murray RM. Structural covariance in the cortex of very preterm adolescents: a voxel-based morphometry study. Hum Brain Mapp. (2011) 32:1615–25. 10.1002/hbm.21133 20853378 PMC6870491

[37] Lax ID Duerden EG Lin SY Chakravarty MM Donner EJ Lerch JP. Neuroanatomical consequences of very preterm birth in middle childhood. Brain Struct Funct. (2013) 218:575–85. 10.1007/s00429-012-0417-2 22572806

[38] Brummelte S Grunau RE Chau V Poskitt KJ Brant R Vinall J. Procedural pain and brain development in premature newborns. Ann Neurol. (2012) 71:385–96. 10.1002/ana.22267 22374882 PMC3760843

[39] Nosarti C Al-Asady MH Frangou S Stewart AL Rifkin L Murray RM. Adolescents who were born very preterm have decreased brain volumes. Brain. (2002) 125:1616–23. 10.1093/brain/awf157 12077010

[40] Nosarti C Giouroukou E Healy E Rifkin L Walshe M Reichenberg A. Grey and white matter distribution in very preterm adolescents mediates neurodevelopmental outcome. Brain. (2008) 131:205–17. 10.1093/brain/awm282 18056158

[41] Vinall J Grunau RE Brant R Chau V Poskitt KJ Synnes AR. Slower postnatal growth is associated with delayed cerebral cortical maturation in preterm newborns. Sci Transl Med. (2013) 5:168ra8. 10.1126/scitranslmed.3004666 23325801

[42] Ranger M Chau CM Garg A Woodward TS Beg MF Bjornson B. Neonatal pain-related stress predicts cortical thickness at age 7 years in children born very preterm. PLoS ONE. (2013) 8:e0076702. 10.1371/journal.pone.0076702 24204657 PMC3800011

[43] Fitzgerald M Beggs S. Book review: the neurobiology of pain: developmental aspects. Neuroscientist. (2001) 7:246–57. 10.1177/107385840100700309 11499403

[44] Schwaller F Fitzgerald M. The consequences of pain in early life: injury-induced plasticity in developing pain pathways. Eur J Neurosci. (2014) 39:344–52. 10.1111/ejn.12414 24494675 PMC4264936

[45] Slater R Fabrizi L Worley A Meek J Boyd S Fitzgerald M. Premature infants display increased noxious-evoked neuronal activity in the brain compared to healthy age-matched term-born infants. Neuroimage. (2010) 52:583–9. 10.1016/j.neuroimage.2010.04.253 20438855

[46] Taddio A Shah V Atenafu E Katz J. Influence of repeated painful procedures and sucrose analgesia on the development of hyperalgesia in newborn infants. Pain®. (2009) 144:43–8. 10.1016/j.pain.2009.02.012 19329255

[47] Andrews K Fitzgerald M. The cutaneous withdrawal reflex in human neonates: sensitization, receptive fields, and the effects of contralateral stimulation. Pain. (1994) 56:95–101. 10.1016/0304-3959(94)90154-6 8159446

[48] Grunau RE Holsti L Haley DW Oberlander T Weinberg J Solimano A. Neonatal procedural pain exposure predicts lower cortisol and behavioral reactivity in preterm infants in the NICU. Pain. (2005) 113:293–300. 10.1016/j.pain.2004.10.020 15661436 PMC1447527

[49] Grunau RE Weinberg J Whitfield MF. Neonatal procedural pain and preterm infant cortisol response to novelty at 8 months. Pediatrics. (2004) 114:e77–84. 10.1542/peds.114.1.e77 15231977 PMC1351380

[50] Cong X Wu J Vittner D Xu W Hussain N Galvin S. The impact of cumulative pain/stress on neurobehavioral development of preterm infants in the NICU. Early Hum Dev. (2017) 108:9–16. 10.1016/j.earlhumdev.2017.03.003 28343092 PMC5444300

[51] Grunau RE Whitfield MF Petrie-Thomas J Synnes AR Cepeda IL Keidar A. Neonatal pain, parenting stress and interaction, in relation to cognitive and motor development at 8 and 18 months in preterm infants. Pain. (2009) 143:138–46. 10.1016/j.pain.2009.02.014 19307058 PMC2836793

[52] Vinall J Miller SP Bjornson BH Fitzpatrick KP Poskitt KJ Brant R. Invasive procedures in preterm children: brain and cognitive development at school age. Pediatrics. (2014) 133:412–21. 10.1542/peds.2013-1863 24534406 PMC3934331

[53] Bucsea O Pillai Riddell R. Non-pharmacological pain management in the neonatal intensive care unit: managing neonatal pain without drugs. In: Boyle E editor. Seminars in Fetal and Neonatal Medicine. Amsterdam: Elsevier (2019). p. 1–6. 10.1016/j.siny.2019.05.009 31326301

[54] Darwin C. The Expression of Emotions in Man and Animals. Chicago: University of Chicago Press (1872).

[55] Horton RE Pillai Riddell R. Mothers' facial expressions of pain and fear and infants' pain response during immunization. Infant Ment Health J. (2010) 31:397–411. 10.1002/imhj.20262 28543081

[56] Horton RE Osmun LD Pillai Riddell R Stevens B Greenberg S. Maternal relationship style, paediatric health care use and infant health. Paediatr Child Health. (2010) 15:432–6. 10.1093/pch/15.7.432 21886447 PMC2948775

[57] Waxman J Martin J Pillai Riddell R. The infant in pain: an attachment perspective on the OUCH Cohort. In: Bellieni C Buonocore G editors. Neonatal Pain Suffering, Pain, and Risk of Brain Damage in the Fetus and Newborn - Second Edition. New York, NY: Springer Publishers (2017). p. 89–104.

[58] Beeghly M Tronick E. Early resilience in the context of parent–infant relationships: A social developmental perspective. Curr Probl Pediatr Adolesc Health Care. (2011) 41:197–201. 10.1016/j.cppeds.2011.02.005 21757137 PMC3137799

[59] Francis DD Diorio J Plotsky PM Meaney MJ. Environmental enrichment reverses the effects of maternal separation on stress reactivity. J Neurosci. (2002) 22:7840–3. 10.1523/JNEUROSCI.22-18-07840.2002 12223535 PMC6758090

[60] Milgrom J Newnham C Anderson PJ Doyle LW Gemmill AW Lee K. Early sensitivity training for parents of preterm infants: impact on the developing brain. Pediatr Res. (2010) 67:330–5. 10.1203/PDR.0b013e3181cb8e2f 19952869

[61] Tu MT Grunau RE Petrie-Thomas J Haley DW Weinberg J Whitfield MF. Maternal stress and behavior modulate relationships between neonatal stress, attention, and basal cortisol at 8 months in preterm infants. Dev Psychobiol. (2007) 49:150–64. 10.1002/dev.20204 17299787 PMC1851900

[62] Vinall J Miller SP Synnes AR Grunau RE. Parent behaviors moderate the relationship between neonatal pain and internalizing behaviors at 18 months corrected age in children born very prematurely. Pain®. (2013) 154:1831–9. 10.1016/j.pain.2013.05.050 23748079 PMC3791108

[63] Franck LS O'Brien K. The evolution of family-centered care: from supporting parent-delivered interventions to a model of family integrated care. Birth Defects Res. (2019) 111:1044–59. 10.1002/bdr2.1521 31115181

[64] Moore KAC Coker K DuBuisson AB Swett B Edwards WH. Implementing potentially better practices for improving family-centered care in neonatal intensive care units: successes and challenges. Pediatrics. (2003) 111:e450–60. 12671165

[65] Gooding JS Cooper LG Blaine AI Franck LS Howse JL Berns SD. Family support and family-centered care in the neonatal intensive care unit: origins, advances, impact. Semin Perinatol. (2011) 35:20–8. 10.1053/j.semperi.2010.10.004 21255703

[66] Melnyk BM Feinstein NF. Reducing hospital expenditures with the COPE (Creating Opportunities for Parent Empowerment) program for parents and premature infants: an analysis of direct healthcare neonatal intensive care unit costs and savings. Nurs Adm Q. (2009) 33:32–7. 10.1097/01.NAQ.0000343346.47795.13 19092521 PMC7521456

[67] Bastani F Abadi TA Haghani H. Effect of family-centered care on improving parental satisfaction and reducing readmission among premature infants: a randomized controlled trial. J Clin Diagn Res. (2015) 9:SC04–8. 10.7860/JCDR/2015/10356.5444 25738051 PMC4347142

[68] Melnyk BM Feinstein NF Alpert-Gillis L Fairbanks E Crean HF Sinkin RA. Reducing premature infants' length of stay and improving parents' mental health outcomes with the Creating Opportunities for Parent Empowerment (COPE) neonatal intensive care unit program: a randomized, controlled trial. Pediatrics. (2006) 118:e1414–27. 10.1542/peds.2005-2580 17043133

[69] Sullivan RM Landers M Yeaman B Wilson DA. Good memories of bad events in infancy. Nature. (2000) 407:38–9. 10.1038/35024156 10993064 PMC9196994

[70] Olsson E Ahlsén G Eriksson M. Skin-to-skin contact reduces near-infrared spectroscopy pain responses in premature infants during blood sampling. Acta Paediatr. (2016) 105:376–80. 10.1111/apa.13180 26342142

[71] Jones L Laudiano-Dray MP Whitehead K Meek J Fitzgerald M Fabrizi L. The impact of parental contact upon cortical noxious-related activity in human neonates. Eur J Pain. (2020) 25:149–59. 10.1002/ejp.1656 32965725 PMC8436758

[72] Pillai Riddell R Racine NM Gennis HG Turcotte K Uman LS Horton RE. Non-pharmacological management of infant and young child procedural pain. Cochrane Database Syst Rev. (2015) 12:CD006275. 10.1002/14651858.CD006275.pub3 26630545 PMC6483553

[73] Johnston C Campbell-Yeo M Disher T Benoit B Fernandes A Streiner D. Skin-to-skin care for procedural pain in neonates. Cochrane Database Syst Rev. (2017) CD008435. 10.1002/14651858.CD008435.pub3 28205208 PMC6464258

[74] Gomes Neto M da Silva Lopes IA Araujo ACCLM Oliveira LS Saquetto MB. The effect of facilitated tucking position during painful procedure in pain management of preterm infants in neonatal intensive care unit: a systematic review and meta-analysis. Eur J Pediatr. (2020) 179:699–709. 10.1007/s00431-020-03640-5 32222816

[75] Hartley KA Miller CS Gephart SM. Facilitated tucking to reduce pain in neonates: evidence for best practice. Adv Neonatal Care. (2015) 15:201–8. 10.1097/ANC.0000000000000193 26002861

[76] Alemdar DK Özdemir FK. Effects of covering the eyes versus playing intrauterine sounds on premature infants' pain and physiological parameters during venipuncture. J Pediatr Nurs. (2017) 37:e30–6. 10.1016/j.pedn.2017.06.016 28751136

[77] Alemdar DK. Effect of recorded maternal voice, breast milk odor, and incubator cover on pain and comfort during peripheral cannulation in preterm infants. Appl Nurs Res. (2018) 40:1–6. 10.1016/j.apnr.2017.12.001 29579482

[78] Chen YS Tan YJ Zhou LS. Clinical effect of maternal voice stimulation in alleviating procedural pain in hospitalized neonates. Chin J Contemporary Pediatr. (2019) 21:58–63. 10.7499/j.issn.1008-8830.2019.01.011 30675865 PMC7390185

[79] Chirico G Cabano R Villa G Bigogno A Ardesi M Dioni E. Randomised study showed that recorded maternal voices reduced pain in preterm infants undergoing heel lance procedures in a neonatal intensive care unit. Acta Paediatr. (2017) 106:1564–68. 10.1111/apa.13944 28580602

[80] Bergomi P Chieppi M Maini A Mugnos T Spotti D Tzialla C. Nonpharmacological techniques to reduce pain in preterm infants who receive heel-lance procedure: a randomized controlled trial. Res Theory Nurs Prac. (2014) 28:335–48. 10.1891/1541-6577.28.4.335 25577862

[81] Badr LK Demerjian T Daaboul T Abbas H Zeineddine MH Charafeddine L. Preterm infants exhibited less pain during a heel stick when they were played the same music their mothers listened to during pregnancy. Acta Paediatr. (2017) 106:438–45. 10.1111/apa.13666 27883227

[82] Jebreili M Neshat H Seyyedrasouli A Ghojazade M Hosseini MB Hamishehkar H. Comparison of breastmilk odor and vanilla odor on mitigating premature infants' response to pain during and after venipuncture. Breastfeed Med. (2015) 10:362–5. 10.1089/bfm.2015.0060 26252909

[83] Küçük Alemdar D Kardaş Özdemir F. Effects of having preterm infants smell amniotic fluid, mother's milk, and mother's odor during heel stick procedure on pain, physiological parameters, and crying duration. Breastfeed Med. (2017) 12:297–304. 10.1089/bfm.2017.0006 28414516

[84] Efendi D Rustina Y Gayatri D. Pacifier and swaddling effective in impeding premature infant's pain score and heart rate. Enfermeria Clin. (2018) 28:46–50. 10.1016/S1130-8621(18)30035-4

[85] Behnam Vashani H Zeraati H Rezaeian A Abrishami M Reyhani T Shoeibi N. The effects of multi-sensory stimulation on the facial expression of neonates during eye examinations for retinopathy of prematurity screening. J Babol Univ Med Sci. (2015) 17:19–24. 10.22088/JBUMS.17.5.3

[86] Chuang LJ Wang SH Ma MC Lin CN Chen CL Huang MC. A modified developmental care bundle reduces pain and stress in preterm infants undergoing examinations for retinopathy of prematurity: a randomised controlled trial. J Clin Nurs. (2019) 28:545–59. 10.1111/jocn.14645 30091495

[87] Wu H-P Yang L Lan H-Y Peng H-F Chang Y-C Jeng M-J. Effects of combined use of mother's breast milk, heartbeat sounds, and non-nutritive sucking on preterm infants' behavioral stress during venipuncture: a randomized controlled trial. J Nurs Scholarsh. (2020) 52:467–75. 10.1111/jnu.12571 32564489

[88] Gao H Li M Gao H Xu G Li F Zhou J. Effect of non-nutritive sucking and sucrose alone and in combination for repeated procedural pain in preterm infants: a randomized controlled trial. Int J Nurs Stud. (2018) 83:25–33. 10.1016/j.ijnurstu.2018.04.006 29684832

[89] Ho LP Ho SS Leung DY So WK Chan CW. A feasibility and efficacy randomised controlled trial of swaddling for controlling procedural pain in preterm infants. J Clin Nurs. (2016) 25:472–82. 10.1111/jocn.13075 26818372

[90] Dezhdar S Jahanpour F Bakht S F Ostovar A. The effects of kangaroo mother care and swaddling on venipuncture pain in premature neonates: a randomized clinical trial. Iranian Red Crescent Med J. (2016) 18:e29649. 10.5812/ircmj.29649 27274399 PMC4894081

[91] Ranjbar A Bernstein C Shariat M Ranjbar H. Comparison of facilitated tucking and oral dextrose in reducing the pain of heel stick in preterm infants: a randomized clinical trial. BMC Pediatr. (2020) 20:162. 10.1186/s12887-020-2020-7 32290829 PMC7155270

[92] Taplak AS Bayat M. Comparison the effect of breast milk smell, white noise and facilitated tucking applied to Turkish preterm infants during endotracheal suctioning on pain and physiological parameters. J Pediatr Nurs. (2020) 56:e19–26. 10.1016/j.pedn.2020.07.001 32690406

[93] Apaydin Cirik V Efe E. The effect of expressed breast milk, swaddling and facilitated tucking methods in reducing the pain caused by orogastric tube insertion in preterm infants: a randomized controlled trial. Int J Nurs Stud. (2020) 104:103532. 10.1016/j.ijnurstu.2020.103532 32062050

[94] Abbasoglu A Cabioglu MT Tugcu AU Ince DA Tekindal MA Ecevit A. Acupressure at BL60 and K3 points before heel lancing in preterm infants. Explore. (2015) 11:363–6. 10.1016/j.explore.2015.07.005 26242287

[95] Fatollahzade M Parvizy S Kashaki M Haghani H Alinejad-Naeini M. The effect of gentle human touch during endotracheal suctioning on procedural pain response in preterm infant admitted to neonatal intensive care units: a randomized controlled crossover study. J Matern Fetal Neonatal Med. (2020) 1–7. 10.1080/14767058.2020.1755649 32316790

[96] Kristoffersen L Støen R Bergseng H Follestad T Theodorsson E Vederhus B. Skin-to-skin contact during eye examination did not reduce pain compared to standard care with parental support in preterm infants. Acta Paediatr. (2019) 108:1434–40. 10.1111/apa.14699 30561825

[97] Uematsu H Sobue I. Effect of music (brahms lullaby) and non-nutritive sucking on heel lance in preterm infants: a randomized controlled crossover trial. Paediatrics Child Health. (2019) 24:E33–9. 10.1093/pch/pxy072 30792607 PMC6376306

[98] Baudesson de Chanville A Brevaut-Malaty V Garbi A Tosello B Baumstarck K Gire C. Analgesic effect of maternal human milk odor on premature neonates: a randomized controlled trial. J Human Lactation. (2017) 33:300–8. 10.1177/0890334417693225 28346843

[99] Perroteau A Nanquette MC Rousseau A Renolleau S Bérard L Mitanchez D. Efficacy of facilitated tucking combined with non-nutritive sucking on very preterm infants' pain during the heel-stick procedure: a randomized controlled trial. Int J Nurs Stud. (2018) 86:29–35. 10.1016/j.ijnurstu.2018.06.007 29960105

[100] Campbell-Yeo ML Johnston CC Joseph KS Feely N Chambers CT Barrington KJ. Cobedding and recovery time after heel lance in preterm twins: results of a randomized trial. Pediatrics. (2012) 130:500–6. 10.1542/peds.2012-0010 22926182

[101] Campbell-Yeo M Johnston CC Benoit B Disher T Caddell K Vincer M. Sustained efficacy of kangaroo care for repeated painful procedures over neonatal intensive care unit hospitalization: a single-blind randomized controlled trial. Pain. (2019) 160:2580–8. 10.1097/j.pain.0000000000001646 31356452

[102] Chen KL Lindrea KB Quah-Smith I Schmölzer GM Daly M Schindler T. Magnetic noninvasive acupuncture for infant comfort (MAGNIFIC) – a single-blinded randomised controlled pilot trial. Acta Paediatr. (2017) 106:1780–6. 10.1111/apa.14002 28741805

[103] Kristoffersen L Skogvoll E Hafstrom M. Pain reduction in insertion of a feeding tube in preterm infants: a randomized controlled trial. Pediatrics. (2011) 127:1449–54. 10.1542/peds.2010-3438 21536607

[104] Royal College of Obstetricians and Gynaecologists. Perinatal Management of Pregnant Women at the Threshold of Infant Viability (the Obstetric Perspective) Scientific Impact Paper no. 41. London: UK RCOG (2014).

